# Contribution of extracellular vesicles for the pathogenesis of retinal diseases: shedding light on blood-retinal barrier dysfunction

**DOI:** 10.1186/s12929-024-01036-3

**Published:** 2024-05-10

**Authors:** Beatriz Martins, Maria Pires, António Francisco Ambrósio, Henrique Girão, Rosa Fernandes

**Affiliations:** 1 University Coimbra, Coimbra Institute for Clinical and Biomedical Research (iCBR), Faculty of Medicine, Coimbra, 3000- 548 Portugal; 2 University of Coimbra, Institute of Pharmacology and Experimental Therapeutics, Faculty of Medicine, Coimbra, 3000-548 Portugal; 3 University of Coimbra, Center for Innovative Biomedicine and Biotechnology (CIBB), Coimbra, 3004-531 Portugal; 4 Clinical Academic Center of Coimbra (CACC), Coimbra, 3004-561 Portugal; 5 Association for Innovation and Biomedical Research on Light and Image (AIBILI), Coimbra, 3000-548 Portugal

**Keywords:** Retinal degenerative diseases, Diabetic retinopathy, Age-related macular degeneration, Blood-retinal barrier, Extracellular vesicles, miRNA

## Abstract

Retinal degenerative diseases, including diabetic retinopathy (DR) and age-related macular degeneration (AMD), loom as threats to vision, causing detrimental effects on the structure and function of the retina. Central to understanding these diseases, is the compromised state of the blood-retinal barrier (BRB), an effective barrier that regulates the influx of immune and inflammatory components. Whether BRB breakdown initiates retinal distress, or is a consequence of disease progression, remains enigmatic. Nevertheless, it is an indication of retinal dysfunction and potential vision loss.

The intricate intercellular dialogues among retinal cell populations remain unintelligible in the complex retinal milieu, under conditions of inflammation and oxidative stress. The retina, a specialized neural tissue, sustains a ceaseless demand for oxygen and nutrients from two vascular networks. The BRB orchestrates the exchange of molecules and fluids within this specialized region, comprising the inner BRB (iBRB) and the outer BRB (oBRB). Extracellular vesicles (EVs) are small membranous structures, and act as messengers facilitating intercellular communication in this milieu.

EVs, both from retinal and peripheral immune cells, increase complexity to BRB dysfunction in DR and AMD. Laden with bioactive cargoes, these EVs can modulate the retinal microenvironment, influencing disease progression. Our review delves into the multifaceted role of EVs in retinal degenerative diseases, elucidating the molecular crosstalk they orchestrate, and their microRNA (miRNA) content. By shedding light on these nanoscale messengers, from their biogenesis, release, to interaction and uptake by target cells, we aim to deepen the comprehension of BRB dysfunction and explore their therapeutic potential, therefore increasing our understanding of DR and AMD pathophysiology.

## Introduction

Retinal degenerative diseases cast a shadow over vision, disrupting the delicate cellular interplay within the retina’s intricate architecture. Among these diseases, diabetic retinopathy (DR) and age-related macular degeneration (AMD) stand as major causes of vision loss and blindness. A focal point in understanding their pathophysiology lies in the compromised state of the blood-retinal barrier (BRB), an integral safeguard that prevents the intrusion of immune and inflammatory elements. Whether the BRB breakdown initiates the cascade of events that lead to retinal distress or unfolds due to disease progression remains a pivotal enigma. Despite its source, this breakdown indicates potential retinal issues and the imminent risk of vision loss.

Within the complex environment of local inflammation and oxidative stress, the intricate intercellular dialogues and signaling cascades within retinal cell populations remain elusive in their complexity. The retina, an exquisitely specialized neural tissue between the vitreous body and the choroid, is delineated into two prominent strata. The inner neurosensory layer, bordering the vitreous, orchestrates an intricate complexity of neurons, glial cells, and vascular components. Its counterpart, the outer layer, is fortified by the retinal pigment epithelium (RPE), a barrier that also nourishes and strengthens the retina, thus strongly contributing for its physiology.

At the core of its mission, the retina’s pivotal task is the conversion of light into electrochemical signals, dispatched via the optic nerve for the brain´s interpretation [1]. This vital function propels the retina into a realm of elevated metabolic demand, hinging upon a ceaseless supply of oxygen and nutrients from two vascular networks. Intra-retinal capillaries intricately intertwine through the inner layers, meticulously furnishing them. Complementing this architecture, the choriocapillaris, positioned beneath the retina, crafts a dense mosaic of fenestrated capillaries, fulfilling the outer layers of the retina with the life-enabling blend of oxygen and nutrients [2].

Retinal homeostasis is intricately maintained by the BRB, an almost impermeable barrier that governs the exchange of molecules and fluids between the retinal parenchyma and its vascular components. BRB is divided in two facets: the inner BRB (iBRB) and the outer BRB (oBRB). The iBRB, a complex structure including vascular endothelial cells, pericytes, glial cells and neurons, collectively termed the Neuro-Vascular Unit (NVU), undergo subtle adjustments that safeguard tissue viability. On the other hand, the RPE assumes the command of the oBRB, governing the nutrient flux between the choriocapillaris and the photoreceptors layer [3–5]. Key components of the BRB are the tight junctions (TJ), intricate assemblages of over 40 transmembrane and intracellular scaffolds, including claudins, occludin, junctional adhesion molecules (JAMs), and zonula occludens (ZO) proteins [6]. These molecular players cement the structural integrity, with ZO proteins orchestrating the balance between the transmembrane TJ proteins and the actin cytoskeleton [7]. Fostering effective dialogue among contiguous retinal endothelial and epithelial cells, the junctional complex establishes a partnership with extracellular vesicles (EVs) to enhance intercellular communication.

Moreover, the molecular and cellular underpinnings of BRB impairment beckon for elucidation, as they harbor the potential to reveal novel therapeutic targets. EVs, small membranous structures released by cells, have emerged as potent mediators of intercellular communication, transporting a cargo of molecular information across cellular frontiers [8]. By exploring EVs’ genesis, isolation, characterization, and communicative functions, we aim at decoding the intricate language of cellular communication within the retinal milieu.

Furthermore, the contributions of EVs secreted by both retinal and peripheral immune cells add an additional layer of intricacy to the BRB dysfunction, namely in DR and AMD. Laden with bioactive cargoes, these EVs possess the potential to modulate the retinal microenvironment, potentially exacerbating or alleviating disease progression. By scrutinizing the actions of these microscopic messengers, we pave the way toward a deeper understanding of the intricate mechanisms orchestrating the progression of retinal degenerative diseases.

In this review, we aim to uncover the multifaceted role of EVs in retinal degenerative diseases. By shedding light on the molecular crosstalk orchestrated by these vesicles, including not only their protein content but also the microRNAs (miRNAs) they carry, our quest is to unravel the intricacies of BRB dysfunction and explore their potential for innovative therapeutic strategies. Indeed, miRNAs have been found to be deregulated in multiple processes associated with retinal diseases development and can be useful as potential biomarkers for the early disease detection and monitoring of its progression [9, 10]. Moreover, miRNAs can act as important therapeutic agents offering a targeted approach to regulate specific pathways implicated in disease pathogenesis. A comprehensive understanding of EV-mediated disease mechanisms requires exploration of their entire cargo. Some interesting reviews about non-miRNA content in EVs on AMD and DR pathological process have already been published [11, 12]. This review aims to give an overview also of some non-miRNA content, but specially to provide a more in-depth comprehension of the role of miRNA-containing EVs in the pathogenesis of retinal degenerative diseases. Through this, we endeavor to contribute to the broader understanding of the pathophysiology of DR and AMD, with the ultimate goal of sight preservation.

## Extracellular vesicles as carriers of information

### Intercellular communication networks through EVs: biogenesis machinery and subtypes

Intercellular communication networks intricately orchestrate tissue homeostasis within multicellular organisms. This communication occurs through diverse mechanisms, including direct cell-cell interactions, the release of soluble factors, such as cytokines and metabolites, and the secretion of EVs [13, 14]. EVs, lipid-bilayer-enclosed vesicles, have emerged as key players in cell-to-cell communication. Initially regarded as cellular debris with no biological significance, the understanding of EVs has drastically evolved since their initial description in 1983, when two independent groups observed the release of vesicles from reticulocytes [15, 16]. Recent years of extensive research on EVs have underscored their pivotal roles in both normal physiological processes and the pathogenesis of various diseases, including age-related conditions [17].

EVs, produced and secreted by diverse cell types, mirror the molecular composition of their parent cells. Laden with proteins, lipids, and nucleic acids, such as DNA, mRNA, and miRNA, EVs populate a range of body fluids, including serum, plasma, tears, urine, and cerebrospinal fluid [18, 19].

**Fig. 1:**
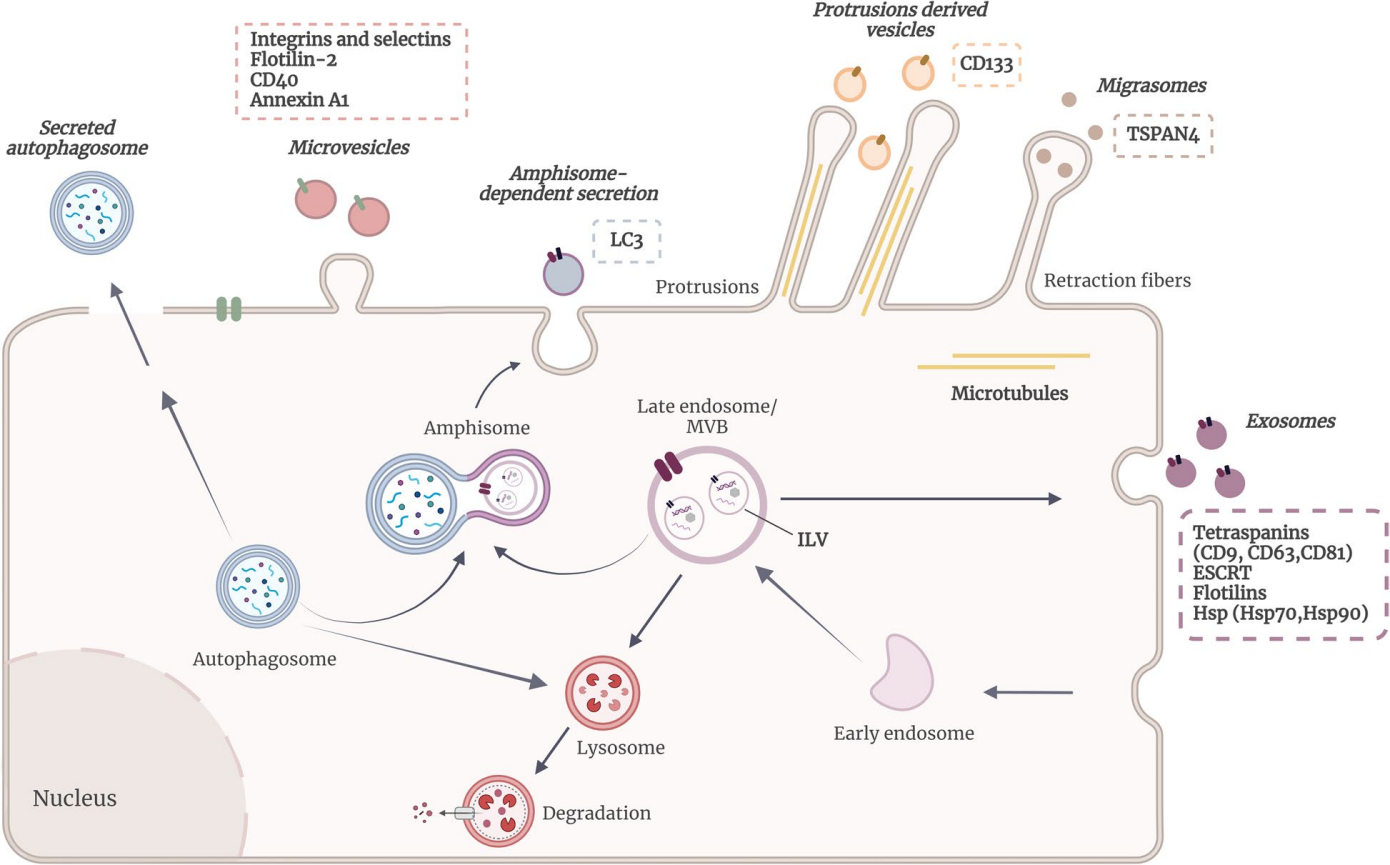
Overview of different subtypes of extracellular vesicles. The various subtypes of extracellular vesicles (EVs) differ from each other in their biogenesis’ pathways and functions. The exosomes are the smallest vesicles, are formed within multivesicular bodies (MVBs) and then released upon MVB fusion with the plasma membrane. Exosomes are enriched with markers such ESCRT-related proteins, tetraspanins or flotillins. Microvesicles bud directly from the plasma membrane and often carry markers like Annexin A1, integrins, and selectins. Protrusion-derived vesicles generate at the tips of membrane protrusions, such as filopodia and microvilli, and often carry CD133 in their content. Migrasomes are a specialized subtype of EVs associated with cell migration that are formed from the disassembly of actin-based membrane protrusions and can be identified by the presence of TSPAN4. Finally, secreted amphisomes and secreted autophagosomes are vesicles originated by the fusion of autophagosomes with MVBs that are released to the extracellular compartment or by the release of autophagosomes, respectively. On both cases, LC3 is a protein commonly associated with these subtypes of EVs. This comprehensive overview of EVs subtypes, illustrated in this schematic representation of a general eukaryotic cell, emphasizes their remarkable diversity and underscores their crucial roles in intercellular communication, contributing to a wide array of physiological and pathological processes. Created with BioRender.com

Classified into two primary groups, according to their size and biogenesis mechanisms, EVs from eukaryotic cells encompass two prominent types of vesicles: exosomes and microvesicles (also known as ectosomes) **(**Fig. 1; Table 1**)**. Exosomes, the smallest vesicles, with a diameter of 30–150 nm, stem from the maturation of early endosomes, generating intraluminal vesicles (ILVs) via inward budding of the endosomal membrane, and then multivesicular bodies (MVBs) formation [20, 21]. ILVs can be generated through the endosomal sorting complex required for transport (ESCRT) machinery that consists of four cytosolic protein complexes (ESCRT-0, I, II, and III) that are recruited to the endosomes by membrane proteins previously ubiquitinated, inducing curvature of the endosomal membrane. These proteins seem to play a role in the budding of ILVs and in the selection of cargo to be loaded into the vesicles [22]. Nevertheless, other ESCRT-independent mechanisms have also been described for the formation of ILVs and selection of cargo, like the neutral sphingomyelinase 2 (nSMase2)-ceramide pathway or the involvement of other proteins, like tetraspanins (CD9, CD63 and CD81), caveolin-1, and flotillins [23, 24]. During this process, cytosolic components are incorporated in the lumen of the vesicles, while endosomal membrane proteins reside in the membrane of the vesicles. MVBs´ fate includes fusion with lysosomes for degradation or with the plasma membrane, liberating ILVs into the extracellular milieu, now designated as exosomes. Mechanisms for MVBs selectivity, however, are not fully understood [25].

Microvesicles are cell membrane-derived vesicles larger than exosomes, ranging from 100 to 1000 nm in diameter. Microvesicles are shed from the surface of a myriad cell types through direct outward budding of the plasma membrane. Increased intracellular calcium levels enhance calpain activation and trigger cytoskeletal protein degradation, promoting localized changes in plasma membrane components. These alterations reshape membrane curvature, culminating in the release of microvesicles into the extracellular space [18, 26, 27].

Other populations of EVs have been reported, such as apoptotic bodies, migrasomes, and amphisomes (Fig. 1; Table 1**)**. Apoptotic bodies, the largest vesicles (50–5000 nm diameter), arise during late-stage apoptosis. These vesicles feature an outer leaflet abundant in phosphatidylserine (PS) and a permeable membrane housing nuclear material, organelles, and membrane/cytosolic components [14, 28, 29]. Despite being less studied than exosomes and microvesicles, apoptotic bodies demonstrate significant biological relevance, regulating processes, such as cell clearance and tissue homeostasis, being also essential to control immune response [30]. Yet, the complete characterization of their molecular composition and role in intercellular communication warrants further exploration.

Migrasomes, distinct in their larger size (up to 3 mm in diameter), serve as vehicles employed by cells to release their cargo, encompassing both smaller vesicles (with diameters around 50–100 nm) and larger vesicles. In addition to conveying cytosolic proteins and mRNA, these vesicles play a crucial role in shuttling damaged mitochondria to the extracellular space, an intricate phenomenon called migracytosis. Within the realms of migrasome biogenesis, these vesicles emerge at the tips or intersections of retraction fibers and are produced as a result of tetraspanin-enriched macrodomain accumulation in the membrane of these fibers, which, resembling elongated tubular strands, manifest as migrating cells retract from the substratum [31, 32].

In secretory autophagy, autophagosomes act as couriers, delivering cargo for unconventional secretion beyond the cell’s boundaries. Autophagy, a protective mechanism, serves to supply nutrients during scarcity and dispose of waste, encompassing damaged organelles and aggregated proteins. This intricate process entails enclosing cargo within autophagosomes - double-membrane vesicles. Subsequently, these autophagosomes fuse either with MVB, forming amphisomes, or directly with lysosomes, facilitating cargo degradation. Beyond its core degradation function, the autophagic machinery is also a key participant in secretory autophagy. Some studies have recently documented this unconventional secretion machinery that drives the fusion of amphisomes with the cell membrane, orchestrating the extrusion of cytoplasmic constituents from the cell. Notably, secretory autophagy has been documented in cells grappling with lysosomal dysfunction, suggesting that this secretion process might be an alternative route for expelling cellular waste materials [33, 34]. In the most recent findings, a novel subtype of vesicles has emerged, originating from extensions covered by the plasma membrane, such as filopodia, microvilli, and cilia **(**Fig. 1; Table 1**)**. These protrusions represent highly dynamic structures that regulate various cellular processes, including motility and adhesion, while also playing a crucial role in intercellular communication by releasing vesicles derived from these protrusions. The shedding of these vesicles appears to be orchestrated by the protein machinery associated with the biogenesis of cellular protrusions and mechanical forces. This subtype of EVs, which can be similar to exosomes or larger, is only beginning to be understood. However, not only mechanical forces but also proteins like Prominin-1 (also known as CD133) and I-BAR domain–containing proteins appear to be involved in the shedding process of these vesicles [35–37].

### The complex landscape of EVs isolation and characterization

EVs have garnered significant interest due to their potential to convey vital intercellular communication. However, unveiling the intricacies of their origin, biological activities, and functions demands precise isolation methods to ensure accurate inference from their contents. Many techniques have been employed to isolate EVs or specific subpopulations, devoid of contaminants, aiming to unveil their roles. However, challenges arise, as most isolation methods primarily capture small EVs, often interchangeably referred as exosomes, by omitting larger vesicles without ascertaining their origin. Additional concerns in EVs isolation include the risk of damage during the process, issues of purity, and low yield. Despite this, several strategies have emerged that capitalize on EVs´ size, density, and solubility, each offering distinct advantages and drawbacks [38, 39].

Differential ultracentrifugation, a widely employed approach, relies on successive rounds of centrifugation cycles at different centrifugal forces and durations to isolate exosomes. Density-gradient centrifugation enhances purity by integrating ultracentrifugation with a density gradient medium (usually sucrose or iodixanol) to increase the purity of the isolated EVs. Another technique, ultrafiltration, is employed either in conjunction with differential ultracentrifugation or independently, harnessing filters of different pore diameters (0.8, 0.45, 0.22, and 0.1 nm) to exclude vesicles beyond the target size range. Size-exclusion chromatography employs porous polymers to separate vesicles based on their hydrodynamic radius. Vesicles larger than the pore of the polymer are eluted first, while smaller vesicles are eluted later, allowing exosome separation. Alternatively, immunoaffinity-based methods use specific antibody-antigen interactions to isolate exosomes by targeting membrane proteins. Precipitation is a simple method that involves mixing biological fluids with a polymer (usually polyethylene glycol (PEG)), followed by lower-speed centrifugation to precipitate exosomes. Commercial kits like ExoQuick™, Invitrogen Total Exosome Isolation Reagent, and miRCURY™ are often employed for this purpose [38–41]. Asymmetric-flow field-flow fractionation (AF4), a recent method based on hydrodynamic size, distinguishes exosome sub-populations. Finally, emerging innovations, such as microfluidic devices, capitalize on specific surface markers or size and density of EVs to facilitate isolation and characterization [42, 43].

Distinguishing between EVs subtypes remains challenging due to overlapping size ranges and the absence of standardized isolation methods. Several studies have identified different markers according to EVs biogenesis pathways [44–46], which helps characterizing the isolated vesicles’ population (Fig. 1; Table 1). Since exosomes originate through the endocytic pathway, their markers are mainly related to MVB formation. The ESCRT proteins ALG-2-interacting protein (Alix) and tumor susceptibility gene 101 (TSG101), as well as the tetraspanins CD9, CD63, and CD81, are the most consensus markers for exosomes, that are usually used in combination to characterize this type of vesicles. Nevertheless, other proteins involved in membrane transport, like annexins and flotillins, class II major histocompatibility complex (MHCII), and heat-shock proteins (HSP), mainly HSP70 and HSP90, are also present in exosomes. In terms of microvesicles, their markers are less well-defined. However, proteins from the ESCRT machinery, annexin A1, flotillin-2, ADP-ribosylation factor 6 (ARF6), matrix metalloproteinases (MMPs), integrins, selectins, and CD40 ligand, as well as lipids such as PS and cholesterol, commonly found on the cell membrane, have been used as markers for microvesicles. The composition of apoptotic bodies remains less understood, with studies noting the enrichment of nuclear material, histones, annexin V and increased levels of PS [29, 47, 48]. Regarding the most recently described EVs, such as migrasomes, secretory amphisomes, and protrusion-derived vesicles, the markers are less well described. However, several studies have demonstrated an effort to characterize these new types of EVs successfully. Migrasomes are known to be enriched in Tetraspanin-4 (TSPAN4) [31]. Regarding secretory amphisomes, the most consensual marker is the microtubule-associated protein 1 A/1B-light chain 3 (LC3) since they are originated through the fusion of an autophagosome, highly enriched in LC3, with an MVB [49]. Finally, the protrusion-derived vesicles are characterized not only by the presence of Inverse Bin – Amphiphysin - Rvs (I-BAR) domain - containing proteins, such as MIM and IRSp53, that are proteins involved in the outward curvature of the plasma membrane, responsible for the formation of these protrusions, but also by the presence of prominin-1 (also known as CD133) [36]. Despite these markers and size differences, the scientific community grapples with identifying markers that adequately distinguish EVs subpopulations and their biological functions [17].

**Table 1:** Comprehensive overview of extracellular vesicle subtypes

	Size	Markers	Biogenesis/release	Refs
Exosomes	30–150 nm	Proteins from ESCRT machinery (Alix and TSG101), tetraspanins (CD9, CD63 and CD81), flotillins, MHCII, HSPs (HSP70 and HSP90), etc.	ILVs formed by inward budding of endosomal membrane (MVB) followed by fusion of the MVB with the plasma membrane	[21]
Microvesicles	100–1000 nm	Proteins from ESCRT machinery, annexin A1, flotillin-2, ARF6, MMPs, integrins, selectins, CD40 ligand, etc.	Outward budding of the plasma membrane	[50, 51]
Apoptotic bodies	50–5000 nm	PS, histones, annexin V and nuclear material	Release during late-stage apoptosis	[28]
Migrasomes	Up to 3000 nm	TSPAN4	Emerge at the tips or intersections of retraction fibers during cell migration	[31]
Secretory autophagy	Unknown	LC3	Autophagosomes release their cargo outside of the cell, mainly in cells with lysosomal dysfunction	[33, 34]
Protrusion-derived vesicles	Unknown	Prominin-1 (also known as CD133) and I-BAR domain–containing proteins	Requires the protein machinery involved on the biogenesis of cellular protrusions and mechanical forces	[36]

## Unraveling the dynamics of EVs release into the extracellular space: the importance of EVs-ECM interactions

Upon being released by the parental cells, EVs enter the extracellular space to ultimately interact with recipient cells in autocrine, paracrine, or endocrine fashions. These vesicles, serving as cargo carriers, navigate intricate journeys, shuttling imperative information and signaling molecules between cells [52–56]. Occasionally, their voyage entails entry into circulation, travelling long distances to reach target tissues. This effort, however, turns them susceptible to macrophage clearance or hepatic detoxification [57]. The mechanisms through which EVs traverse body fluids and overcome biological barriers remain somewhat enigmatic [19].

After leaving the parental cell and before interacting with their target, EVs inevitably need to interact with the extracellular matrix (ECM). The ECM, a physical scaffolding comprising collagens, laminins, fibronectin, and proteoglycans, along with other molecules, is a crucial structure that takes part in several important processes, such as structural support, cell adhesion, and tissue repair and remodeling [58]. The interaction of EVs with ECM is a dynamic process that depends on several factors, including the size and composition of the vesicles, as well as the composition and complexity of the ECM. This prompts contemplation on whether differences in ECM composition, mechanical properties, and topographical arrangement in ECM play a role in modulating this capability [59]. Equally intriguing is the observation that EVs manage to penetrate the specialized endothelial ECM, the basement membrane, despite its greater thickness, while avoiding penetration of the epithelial ECM [60]. Nevertheless, this interaction can serve only to allow EVs to travel through the ECM or, in several cases, the EVs play an active role in the modulation of ECM through direct EVs - ECM interactions or by influencing cell - ECM interactions [61].

In health conditions, the majority of our understanding of the interaction between EVs and ECM is based on the well-established roles of matrix vesicles, i.e., EVs released by bone cells that play a critical role in the process of mineralization within developing bones and cartilage [62]. In this case, the EVs become residents of the ECM. However, much less is known about ECM-resident EVs in soft matrices, such as the brain and the eye.

What is well understood is the ability of EVs to transverse the ECM to achieve their target tissue. The direct action of EVs in the ECM actively shape ECM architecture and structure through the action of enzymes present in the surface of EVs, like aggrecans, MMPs, elastase, and possibly other proteases involved in ECM remodeling [63, 64]. Additionally, some EVs may carry specific surface molecules, such as integrins or proteoglycans, facilitating their interaction with ECM components, which can then support the EVs’ attachment to the ECM. The presence of MMPs facilitates the creation of pathways through the ECM, enabling EVs movement [64, 65].

This journey of EVs is essential for their roles in physiological processes, tissue homeostasis, and disease contexts, including tissue repair and tumor development [65, 66]. Their ability to interact with ECM components and modify the ECM allows them to navigate through this dynamic environment. However, to transverse the ECM, these vesicles face various challenges. Understanding the mechanisms by which EVs interact with and traverse the ECM is an active area of research with significant implications for both basic biology and clinical applications.

### Dynamics of EVs interactions and communication

EVs emerge as pivotal orchestrators of intercellular communication, facilitating the transfer of an array of cargoes, including proteins, nucleic acids, and lipids. A defining hallmark of EVs lies in their remarkable ability to target specific cells and adeptly release their cargo, underscoring their immense potential across diverse biological processes, such as cell adhesion, migration, proliferation, differentiation, reprogramming, apoptosis, and immunity [67–73].

Having successfully reached their targets, EVs stand at the precipice of internalization, poised to engage recipient cells by various means. The internalization of EVs manifests through various mechanisms - endocytosis, phagocytosis, macropinocytosis, or even direct fusion with the plasma membrane. The choice of internalization depends on EV subtype, cargo composition, and the recipient cell type [74–77].

Numerous factors interfere with EVs uptake, such as size, shape, surface markers, cell type, microenvironment, exposure time, and dose. The dimensions and shape of EVs dictate their internalization mechanism. Smaller, rounder EVs tend to favor clathrin-mediated endocytosis [78–80]. Conversely, larger EVs, specifically apoptotic bodies, follow a distinct pathway. These vesicles are engulfed by phagocytes through recognition by surface molecules as annexin V, C3b, and thrombospondin on their surface [81]. Additionally, surface markers on EVs also hold sway, as markers like PS tilt the balance towards phagocytic uptake [82]. The microenvironment surrounding the cells adds yet another layer of complexity, with specific ECM components and cytokines promoting specific internalization mechanisms [83].

Significant insights into the traversal of barriers have been gleaned from research on the blood-brain barrier (BBB) [84]. It has been elucidated that EVs employ three distinct endocytosis processes - receptor-mediated transcytosis, lipid-raft-mediated endocytosis, and micropinocytosis - to effectively traverse the BBB [85]. Although numerous candidates, integrins (a5 and aV) and the cluster of differentiation described as an adenovirus receptor CD46, have been pointed as potential receptors of exosomes on endothelial cell surface. Also, EVs exhibit integrins and tetraspanins for specific receptors in recipient cells, such as transferrin and transferrin receptor [84]. In healthy scenarios, the presence of the TJ between endothelial cells restricts paracellular transport. Nonetheless, under specific conditions that lead to the release of exosomes with different compositions, these exosomes can promote vascular permeability by compromising the integrity of the TJ complexes. Consequently, EVs gain the ability to access the circulatory system via the paracellular pathway [86]. Despite our current limited understanding of the mechanisms underlying EVs traversal of the BRB, it is plausible to speculate that they can traverse both from the retina to the blood and vice versa, mainly through transcytosis in a healthy environment and via the paracellular pathway under pathological conditions, thus expanding their functional repertoire within this context.

The intricate targeting of recipient cells by small EVs (sEVs) constitutes a multifaceted and dynamic process encompassing multiple mechanisms. Surface molecules on EVs orchestrate specific interactions with recipient cells, ensuring precise delivery of cargo to designated destinations. These EVs cargoes hold a broad spectrum of bioactive molecules with the potential to modulate cellular functions and actively take part in physiological and pathological processes. Intriguingly, EVs’ secretion and cargo composition exhibit context-dependent alterations, rendering them invaluable indicators for various diseases [87, 88]. Understanding the regulatory mechanisms that govern EVs in intercellular communication is crucial for their clinical applications. The effectiveness of EVs in modulating cellular functions hinges on their interactions with recipient cells. For instance, a study by Liu et al. sheds light on the mechanism through which retinal pericytes and endothelial cells communicate [89]. They demonstrated how diabetes disrupts the crosstalk mediated by sEVs between vascular pericytes and endothelial cells. This communication is essential for microvascular stabilization and remodeling. The study reveals that elevated circular RNA cPWWP2A levels in pericytes under diabetes-induced stress directly affect pericyte biology and indirectly influence endothelial cell biology via exosomes containing cPWWP2A [89], highlighting the significance of EVs uptake in mediating the transfer of signaling molecules and functional cargo, spotlighting the potential therapeutic potential of manipulating this process in disease contexts.

Exploration of EVs in the retinal milieu has been extensively pursued through in vitro research using primary cultures, transformed cell lines, and pluripotent stem cell-derived retinal cell types. These endeavors have scrutinized EVs` composition and biological functions within the retinal context [90–95]. Notably, a study delved into the immunomodulatory effects of RPE cells (using the human RPE cell line ARPE-19) via EVs secretion. These vesicles effectively induce an immunoregulatory CD14 + + CD16 + + phenotype in human monocytes, inhibiting T-cell proliferation and rendering monocytes immunosuppressive under homeostatic conditions, all without impacting their survival [91].

The process of EVs targeting involves a symphony of mechanisms, chiefly driven by molecular interactions between the surface molecules of EVs surface and receptors residing on the target cells. These EVs bear diverse surface molecules, including proteins, lipids, and carbohydrates. Functioning as ligands, these molecules recognize and bind to receptors on the surface of target cells [96]. Among the main groups of surface molecules participating in this complex choreography are integrins, which are transmembrane proteins that play key roles in cell adhesion and signaling. Integrins, with binding sites tailored for specific ligands, facilitate the attachment of EVs to their designated target cells. For instance, EVs derived from oxidatively stressed donor RPE cells may express integrins that engage in specific interactions with integrin receptors on recipient RPE cells. This interaction triggers a captivating sequence, promoting EV binding and clathrin-dependent endocytosis [97]. Tetraspanins are another group of surface molecules on EVs contributing to their targeting abilities. These transmembrane proteins merge into tetraspanin-enriched microdomains (TEMs) on the EVs surface that initiate intricate dialogues, involving other proteins on the EV membrane and receptors on target cells, thereby facilitating EV binding and uptake. Prominent players like CD9, CD63, and CD81, commonly used as markers for EV isolation, take part in the symphony of biological processes, including EVs targeting [98].

Selectins, such as intercellular adhesion molecule 1 (ICAM-1), are a class of cell adhesion molecules that also participate in the nuanced process of EVs targeting. Selectins are pivotal in the initial tethering and rolling of EVs along the endothelial surface. This engagement allows EVs to interact with specific receptors on target cells. This interaction results in immune cell recruitment and inflammation. Immune cell-released EVs interact with selectins on endothelial cells, setting the stage for their targeting towards specific tissues [99, 100].

The temporal dimension cannot be overlooked, with both dosage and exposure time impacting EVs uptake. Higher doses and prolonged exposure lead to augmented uptake [101, 102]. Having traversed this labyrinthine journey, internalized EVs unveil their cargo inside recipient cells. This cargo release affects cell signaling, gene expression, and various cellular functions, often influencing immune response modulation, tissue regeneration, and disease pathogenesis [103–105]. Remarkably, EVs can transmit their information through interaction with receptors/ ligands on the cell surface, triggering a cascade of specific signaling events, all without crossing the cell membrane [55, 106].

Understanding the intricate mechanisms governing the interaction and communication of EVs with recipient cells stands as a fundamental milestone in comprehending their functionality. As we gain insights into how EVs engage with cells, a tapestry of specific roles and functions within cellular processes comes to light. This comprehensive understanding lays the foundation for the development of diverse clinical applications, diagnostics, and drug delivery systems.

EVs play a pivotal role in retinal degenerative diseases, serving as crucial mediators in intercellular communication within the complex microenvironment of the retina. In age-related macular degeneration and diabetic retinopathy, these vesicles contribute to the progression of pathological processes influencing cellular responses and the local microenvironment within the retina.

## Age-related macular degeneration (AMD)

AMD is a prevalent degenerative disease affecting the retina’s macula, causing prominent central vision loss in the elderly. AMD’s phenotypic manifestations are largely linked to RPE dysfunction [107]. Early stages of AMD feature extracellular deposits (drusen) positioned between RPE and Bruch’s membrane, a multilayer membrane separating the RPE from the choriocapillaris. Drusen appearance with age may stem from residual RPE material buildup and Bruch’s membrane alterations, including permeability and thickness changes, leading to extruded material accumulation [108–110]. Progressing to advanced stages, AMD manifests as dry or wet forms, both associated with progressive visual impairment. Dry AMD, or atrophic AMD, involves RPE geographic atrophy and outer retinal thinning, which is associated with oBRB breakdown without neovascularization. Wet AMD, also known as the neovascular form, is characterized by choroidal neovascularization (CNV) and subretinal fluid accumulation or hemorrhage [111].

With AMD being a common retinal degenerative disease in the elderly, persistent oxidative stress has a main role in disease onset. Oxidative stress may have different origins including light exposure, hypercholesterolemia, and smoking [112]. The stress occurs primarily in the RPE, where photoreceptor outer segments, rich in polyunsaturated fatty acids that, undergo digestion, thus inducing reactive oxygen species (ROS) production. Chronic low-grade inflammation also significantly contributes to AMD progression by promoting the accumulation of cellular debris, formation of drusen, dysfunction and death of RPE cells, ultimately leading to geographic atrophy and CNV.

Inflammatory cells, such as macrophages and microglia, infiltrate the retina and choroid in response to chronic inflammation, releasing pro-inflammatory cytokines and chemokines, such as interleukin-6 (IL-6), interleukin-8 (IL-8), tumor necrosis factor-alpha (TNF-α), and complement factors, that induce oxidative stress and apoptosis in RPE cells [113, 114]. Dysregulation of complement activation in AMD further exacerbates inflammation, oxidative stress, and tissue damage, driving retinal degeneration and vision loss in AMD. Notably, variants of the complement factor H (CFH) and other complement components, such as C2, C3, and C5, are implicated in AMD development [115].

In addition to its impact on the RPE, inflammation alters choroidal blood flow, leading to hypoxia and oxidative stress in the outer retina. This in turn, promotes the formation of angiogenic factors, such as vascular endothelial growth factor (VEGF), driving the development of neovascular AMD [116]. The interplay between oxidative stress and inflammation exacerbates retinal damage, perpetuating disease progression in AMD.

While AMD is not primarily considered an inflammatory disease, research suggests the potential efficacy of anti-inflammatory drugs in its treatment. Corticosteroids, for instance, inhibit choroidal endothelial cell migration and tube formation, potentially slowing down the progression of the disease [117]. Additionally, low-dose aspirin or cyclooxygenase-2 (COX-2) inhibitors have shown protective effects against AMD [118]. Combined therapies involving dexamethasone, photodynamic therapy (PDT), and anti-VEGF agents have also demonstrated promise in stabilizing visual acuity [119–121]. Recently approved drugs targeting complement inhibition, such as pegcetacoplan and avacincaptad pegol, offer novel avenues for dry AMD/geographic atrophy [122, 123]. Given the substantial evidence implicating inflammation in AMD, targeting inflammatory pathways remains a logical approach for its management.

### Interplay of oxidative stress and inflammation in AMD: EV-mediated crosstalk

**Fig. 2:**
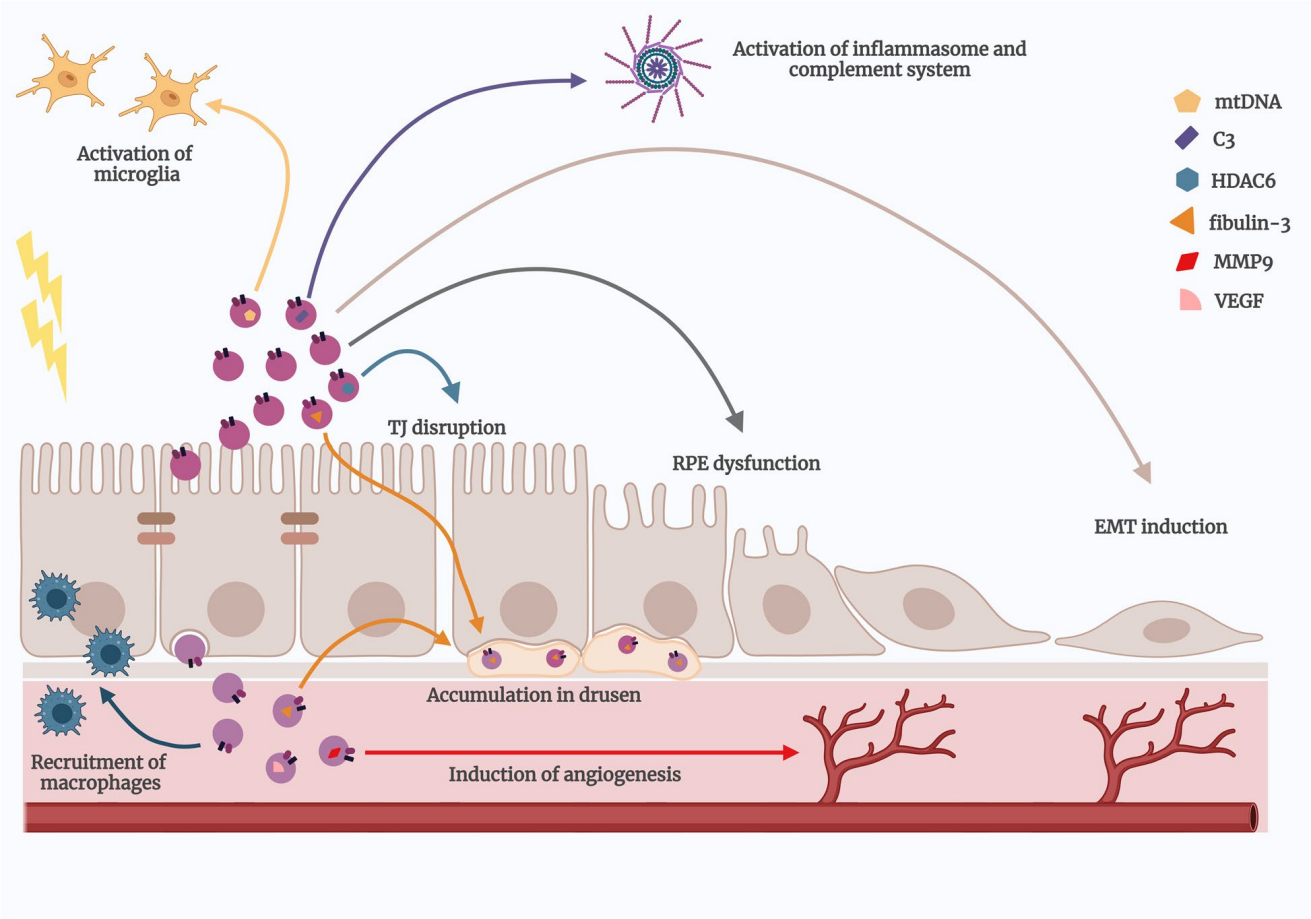
Contribution of extracellular vesicles to age-related macular degeneration pathogenesis. The extracellular vesicles (EVs) have a multifaceted role in the pathogenesis of age-related macular degeneration (AMD), being central players in several critical processes contributing to AMD progression. It is described the presence of EVs co-localized with fibulin-3 within drusen deposits, a classical hallmark of AMD, highlighting their involvement in the disease’s early stages. Once submersed within an oxidative stress and pro-inflammatory environment, retinal pigment epithelial (RPE) cells release EVs that can contribute to the progression of the disease. These RPE-derived EVs are involved in the activation of the immune system, since EVs carrying damaged mitochondrial DNA (mtDNA) have the potential to activate microglia. Their involvement in the activation of the immune system is also related to their ability to participate in the recruitment of macrophages to the site of AMD lesions. Along with these, RPE-derived EVs that carry complement 3 (C3) also activate the inflammasome and the complement system, leading to the release of pro-inflammatory cytokines which consequently contributes to the inflammatory milieu of the AMD microenvironment. Moreover, these EVs can also act in an autocrine manner, being directly involved not only in the disruption of tight junction (TJ) complexes and in the breakdown of the outer blood-retinal barrier (oBRB), carrying histone deacetylase 6 (HDAC6), but also in the dysfunction of other RPE cells and in the induction of the endothelial-mesenchymal transition (EMT) that often occurs in the RPE monolayer, leading to subretinal fibrosis and the formation of hyperreflective foci present in AMD patients. Ultimately, RPE-derived EVs are also implicated in angiogenesis of choroidal vessels, a pivotal event in wet AMD pathogenesis, since they carry pro-angiogenic cargo such as vascular endothelial growth factor (VEGF) and matrix metalloproteinase 9 (MMP-9). All of these events highlight the pivotal role of EVs in driving various AMD-related processes, making them attractive targets for therapeutic interventions in the management of AMD. Created with BioRender.com

Being RPE the primary site of AMD damage, susceptibility across the macula triggers atrophy. Eventually, when the harm is triggered in a susceptible region of the RPE, this may spread to healthy neighboring cells, and enhanced EVs release from stressed cells seem to play a role in this process [124]. EVs may play a role in the pathophysiology of AMD by modifying communication between different cell types (Fig. 2). For instance, EVs could be significantly contributing to paracrine signaling, potentially influencing RPE-mediated dysfunction in photoreceptors (apical) and choroidal dysfunction (basal), in AMD. A study conducted by Klingborn et al. demonstrated that RPE cells secrete exosomes in a polarized manner, with apical EVs having a different proteomic composition than basolateral EVs. This suggests that these EVs have polarity-specific functions that can impact the pathophysiology of AMD [92]. In fact, several studies have been focused on the directional secretion of RPE-derived EVs. For instance, it has been shown that besides the oBRB breakdown, severe oxidative stress leads to the release of αB crystallin, a heat shock protein, via small EVs. αB crystallin is secreted in RPE-derived EVs to the apical site under normal conditions, but when the RPE cells are exposed to severe oxidative stress, these αB crystallin-containing EVs are released to the basolateral side of RPE cells as well [90], which may explain the involvement of RPE-derived EVs on extracellular deposits and RPE atrophy in AMD. A recent study also reported that RPE cells display a directional release of drusen-associated proteins in EVs and that this directionality is susceptible to modulation in response to chronic oxidative stress, which may demonstrate a causal role of RPE-secreted exosomes in promoting AMD pathophysiology [95].

Despite the molecular mechanisms involved in AMD not being completely elucidated, several studies have demonstrated that deregulation of proteolytic pathways, namely chronic inhibition of the proteasome and autophagy activation, can lead to increased exocytic activity with the release of proteins, lipids, and miRNAs via EVs, that contribute to AMD pathogenesis [125]. This can be evidenced by RPE stress induced by lipid accumulation, hindering exosome efflux across Bruch’s membrane and promoting EV´s release linked to drusen accumulation [125, 126]. Markers like CD63 and annexins on drusen reinforce EVs involvement [127]. Fibulin-3, a glycoprotein that is expressed in the retina, is accumulated within EVs in drusen, suggesting that Alix-positive EVs carrying fibulin-3 may be associated with drusen accumulation and play a pathological role in AMD [128]. In a recent study, an immunoaffinity filter paper platform coated with anti-CD63 antibodies as the capture molecule, was used to isolate exosomes from human aqueous humor. This was combined with scanning electron microscopy and microfluidic systems to investigate the size of exosomes in patients with different retinal diseases related to neovascularization, including AMD. The study revealed a higher distribution of exosomes with less than 50 nm in diameter, known as exomeres, which are known to play a significant role in the pathophysiology of ischemic retinal diseases. These exomeres were shown to be enriched in proteins involved in metabolism, coagulation and hypoxia [129].

Recent insights propose dysfunctional RPE cells and microglia EV-mediated crosstalk engendering retinal proinflammation (Fig. 2) [130, 131]. It has been shown that exposure of polarized RPE cells to chronic oxidative stress, a type of insult that is considered to drive AMD, induces significant mitochondrial DNA (mtDNA) damage. The binding of oxidatively damaged mtDNA to DNA receptor Z-DNA-binding protein 1 (ZBP1) in the cytosol of microglial cells leads to the activation of the mtDNA/ZBP1 pathway. Concurrently, EVs containing fragments of mtDNA derived from the apical site of RPE cells are released into the retina that induce a proinflammatory phenotype of microglia, sustaining inflammation that impacts photoreceptors, ganglion, and RPE cells viability. Therefore, activation of the mtDNA/ZBP1 pathway in microglia, induced by mtDNA-containing EVs derived from RPE, may potentially drive the development of chronic inflammation in AMD [132].

In addition to oxidative stress, the complement system is vital in AMD pathogenesis (Fig. 2). EVs carrying inflammatory cargos fuel inflammation and innate immunity in AMD. In fact, some studies already reported the presence of specific complement proteins, including Complement 3 (C3), and AMD-associated proteins, like CD46 and CD59, that play a crucial role in complement regulation in RPE-derived EVs [95, 133].

The decreased levels of membrane-bound complement regulators in RPE cells during AMD might arise from the association of these complement regulators to EVs released in AMD [133, 134]. Moreover, mutations in the CFH gene, strongly associated with an increased risk of AMD, lead to C3-coated exosomes released from RPE cells triggering an immune cell response. This cascade of events results in the destabilization of exosome membranes and the accumulation of intracellular proteins in drusen [127].

It has also been shown that photooxidative RPE damage by blue light exposure boosts exosome secretion, enriched in proteins associated with ECM and drusen, and these vesicles can modulate the NLRP3 inflammasome activity in vitro [135]. These results suggest that RPE-derived EVs are implicated in AMD´s innate immune response. As previously discussed, EVs are one of the most important vehicles involved in cell communication. Furthermore, depending on their function, different cells secrete different EVs with a particular cargo. Particularly in the retina, the communication between all retinal layers is extremely important to maintain the normal vision cycle. One of the most important regulators of cell-cell communication is the BRB, which controls the communication between the retina and the peripheric tissues.

Macrophages have been associated to areas of Bruch’s membrane breakdown, RPE atrophy, and choroidal neovascularization, in AMD patients [113, 136–138]. A shift in macrophage polarization and an increased presence of retinal/ choroidal macrophages have also been consistently associated with CNV [139–142]. These macrophages, which are rich sources of TNF-α, are known to activate RPE cells, by triggering monocyte chemotactic protein (MCP-1) production by RPE and subsequent macrophage recruitment to the subretinal space. In fact, a study reported an increase in CD63-positive EVs in co-cultures of RAW 264.7 macrophages with mouse primary RPE (mpRPE) cells. These EVs were mainly produced by mpRPE cells causing an increased secretion of cytokines, MCP-1, and VEGF. This suggests that RPE-derived EVs actively participate in the sub-retinal innate inflammation by recruiting and activating macrophages (Fig. 2) [143].

Another extremely important EVs-mediated crosstalk during AMD pathogenesis is between RPE and endothelial cells. Several studies have identified angiogenic proteins, such as VEGFA and MMP-9, in RPE-secreted exosomes under inflammatory and oxidative stress conditions. These EVs appear to play an important role in the development of CNV by inducing endothelial cell proliferation and migration and consequently promoting angiogenesis in AMD models (Fig. 2) [93, 124].

### Role of EVs in AMD: insights into RPE and oBRB dysfunction, and inflammation

The RPE, a key component of the oBRB, plays a pivotal role in preserving vision. AMD primarily affects RPE cells, and mounting evidence implicates oxidative stress and inflammation in RPE dysfunction, including oBRB permeability changes. In recent years, the involvement of EVs, particularly those from RPE cells, has unveiled in exacerbating RPE dysfunction in an autocrine manner (Fig. 2). In fact, a recent study demonstrated that EVs released from RPE cells undergoing oxidative stress can communicate stress messages to healthy RPE cells, being able to impair the integrity of TJ, leading to reduced barrier function in the RPE monolayer, as evidenced by decreased transepithelial resistance (TER) [112]. This decrease in the TER can be explained by dynamin-mediated EVs uptake, driven by desialylated glycans and integrin receptors on recipient RPE cells. Notably, stress-induced EVs exhibit heightened ligands for these glycans and receptors, along with neuraminidase activity [97]. A parallel study observed increased permeability in recipient RPE monolayers upon exposure to EVs from RPE cells exposed to oxidative stress. Proteomic analysis revealed histone deacetylase 6 (HDAC6), a known disruptor of TJs, within these EVs. The application of HDAC6 inhibitors mitigated the impact of EVs on RPE cells, underscoring its role in RPE dysfunction [112]. Furthermore, investigations about microvesicles derived from oxidative stress-exposed RPE cells reported reduced RPE cell viability, accompanied by elevated expression of cyclin-dependent kinase inhibitors p15 and p21, markers of senescence. Microparticles also increased senescence and decreased phagocytic activity of RPE cells. These findings emphasize the ability of EVs to exacerbate oxidative stress-induced damage to RPE cells [144]. Consistently, a recent study showed that the exposure of control iPSC-derived RPE to EVs derived from apical RPE exposed to AMD conditions, induce the acquisition of AMD features, namely stress vacuoles, cytoskeletal destabilization and abnormal morphology of the nucleus in control iPSC-RPE cell, along with a disruption of the neuroepithelium in retinal organoids. These results confirm the role of RPE-derived EVs as potent inducers of RPE dysfunction [145].

One of the main features of AMD pathogenesis is the epithelial-mesenchymal transition (EMT) of RPE cells, that is believed to be associated not only with subretinal fibrosis but also with hyperreflective foci (HRF), commonly found in optical coherence tomography (OCT) retinal images of AMD patients. The researchers believe that these HRF, detected in the inner layers of the retina, are mainly RPE cells that undergo EMT and gained the capacity to migrate to the neuroretina [146]. The role of EVs in mediating EMT of RPE cells is a research area that still needs to be more explored. However, a study conducted by Zhang and colleagues demonstrated that EVs secreted by RPE treated with transforming growth factor beta 2 (TGFß-2), a potent inducer of EMT, present an enrichment of EMT-related contents and significantly induced the EMT on RPE recipient cells (Fig. 2) [94].

While understanding the impact of EVs/exosomes secreted by RPE cells on healthy RPE cells, and their role in oBRB function, remains a burgeoning area of research, it is evident that stress-exposed RPE cells release EVs teeming with proteins and miRNA (reviewed below), which are capable of affecting healthy RPE cells and compromising their function. Moreover, these EVs can engage with immune cells, particularly macrophages, potentially triggering a self-perpetuating cycle that not only contributes to RPE dysfunction but also fosters a chronic inflammatory subretinal environment. This intricate interplay highlights the multifaceted role of EVs in AMD pathogenesis and RPE dysfunction (Fig. 2).

## Role of miRNA on AMD pathogenesis

miRNAs are a class of small single-stranded non-coding RNA molecules that repress gene expression at a post-transcriptional level. In AMD, it has been widely shown that changes in miRNA levels are associated with disease onset and/ or progression. miRNAs have been implicated in diverse key aspects of AMD, including autophagy, inflammation, oxidative stress, angiogenesis, apoptosis, and phagocytosis [147]. For this reason, miRNAs have emerged as potential powerful diagnostic and/ or therapeutic tools. The miRNA-mediated regulation of protein synthesis can occur within cells producing the miRNAs or in distant acceptor cells. In this case, miRNAs preferentially use EVs as carriers to mediate long range communication. This strategy is particularly important because EVs protect miRNAs against degradation by RNases present in plasma and body fluids, thus increasing miRNA stability, and it can also contribute to direct miRNA to target cells. Although miRNAs are the most abundant molecules in EVs, their sorting is subjected to fine-tuned processes. Indeed, the selective sorting of miRNA into EV has been reported to be associated with different mechanisms, namely those involving the sphingomyelinase 2 [148], nuclear ribonucleoprotein hnRNPA2B1 [149], miRISC complex [149], 3′-end sequence of miRNA [150] and Cx43 [151]. Landmark studies by Elbay and colleagues compared the miRNA profile of circulating EV of controls and AMD patients and unveiled that miR-486-5p and miR-626 were upregulated in AMD patients, whereas miR-885-5p had lower expression [152]. Since these miRNAs are implicated in apoptosis and neovascularization pathways, it is suggested that they can contribute to wet AMD (Table 2). In agreement with this hypothesis, Atienzar-Aroca and colleagues uncovered that EV secreted by RPE cells exposed to stress are enriched in VEGFR and VEGFR mRNA that can promote angiogenesis when taken up by endothelial cells [124]. Besides angiogenesis, another study reported that EV-enclosed miRNAs associated with AMD can also induce apoptosis of RPE [153]. On the other hand, the intravitreal administration of EVs produced by human umbilical cord-derived mesenchymal stem cells (HucMSCs) ameliorates subretinal fibrosis, in a laser-induced choroidal neovascularization model [154]. This report unraveled that this anti-fibrotic effect of HucMSC-derived EVs relies on miR-27b-3p that, through the downregulation of Homeobox C6 (HOXC6), mediates the suppression of EMT of RPE implicated in subretinal fibrosis.

A study has shown that miR-494-3p present in EVs produced by iPS-derived RPE is able to elicit an inflammatory response by macrophages [155]. Corroborating this evidence, a recent study revealed that the lncRNA CYLD-AS1 can impact on the inflammatory profile of EVs secreted by RPE, namely by sponging miR-134-5p. The knockdown of CYLD-AS1 in ARPE-19 cells results in EV secretion that will further protect RPE cells against inflammation [156]. ARPE-19 cells exposed to hydrogen peroxide, simulating oxidative stress, exhibited increased EVs release, carrying over 200 miRNA, including miR-302a and miR-122. However, miR-302a and miR-122 were downregulated in EVs from stressed RPE cells, potentially linked to neovascularization induced by EVs under oxidative conditions [157]. Moreover, mice exposed to photo-oxidative damage, known as an animal model of dry AMD, exhibit a decrease in the levels of miR-124-3p, which is known to be present in small EVs and play a key role in AMD pathogenesis. A decrease in the levels of this miRNA, especially present in the outer nuclear layer, leads to exacerbated retinal damage, immune cell activation and photoreceptor cell death [158–160].

Altogether, these studies demonstrate the role of miRNAs as important mediators of RPE dysfunction, immune system activation, and neovascularization, among others (Table 2). Together, these findings underscore the significant impact of miRNAs within EVs on AMD pathogenesis, offering potent diagnostic and therapeutic avenues for this complex eye disorder.

**Table 2:** Key miRNAs in extracellular vesicles associated with age-related macular degeneration

miRNA	Expression level in EVs (AMD phenotype)	Predicted target	Function	Ref
miR-486-5p	upregulated	Insulin-like growth factor 1 (IGF-1)/AKT/mTOR pathway and CD40 (TNFRSF5) pathway	Apoptosis, inflammation and neovascularization	[152]
miR-626	upregulated	SLC7A5	Retinal degeneration	[152]
miR-885-5p	downregulated	Insulin like growth factor-1 (IGF-1)	Neovascularization	[152]
miR-27b-3p	downregulated	HOXC6	EMT	[154]
miR-124-3p	downregulated	Chemokine (C-C motif) ligand 2 (Ccl2)	Retinal damage, immune cell activation and photoreceptor cell death	[158–160]
miR-494-3p	upregulated		Inflammatory response of macrophages (enhances M1 macrophage polarization)	[155]
miR-134-5p	upregulated	NRF2/NF-κB signaling pathway	Oxidative stress-related and inflammatory function of RPE cells	[156]
miR-302a and miR-122	downregulated	VEGFA and VEGFC, respectively	Neovascularization	[157]

### Diabetic retinopathy (DR)

DR stands as a prevalent neurovascular complication of diabetes, with an unfortunate status as the leading cause of vision loss among the working-age population in developed countries. DR is marked by a progressive transformation of the retinal vasculature accompanied by neuroglial damage. Notably, around one-third of individuals with diabetes experience some form of this disease, with a higher prevalence noted in those with Type 1 Diabetes Mellitus (T1DM) [161]. DR typically evolves through distinct stages, characterized as non-proliferative DR and proliferative DR [162, 163]. In its initial phases, DR remains asymptomatic, with its diagnosis primarily reliant on observable retinal changes and retinal neovascularization. The mild non-proliferative form of the disease is characterized by the presence of a few microaneurysms associated with localized proliferation of endothelial cells and loss of pericytes. Subsequently, the disease advances into the moderate non-proliferative phenotype, marked by increased number of microaneurysms, small hemorrhages, exudates, and intra-retinal microvascular anomalies (IRMAs). These developments lead to heightened vascular permeability and endothelial cells and pericyte apoptosis, ultimately culminating in iBRB breakdown. Notably, iBRB abnormalities, detectable through fluorescein angiography and vitreous fluorimetry, represent a hallmark of DR. Disease progression then escalates into severe DR, characterized by ‘cotton-wool’ spots, manifestations of retinal ischemia that result in capillary closure and the release of pro-inflammatory and angiogenic factors. Proliferative DR, the most advanced stage, arises when a disruption in the delicate equilibrium between angiogenic factors, particularly VEGF, and anti-angiogenic factors results from severe hypoxia. This imbalance fosters neovascularization, culminating in the growth of fragile vessels extending into the vitreous, leading to vitreous hemorrhage and retinal detachment. Ultimately, this progression results in vision loss [164, 165]. Diabetic macular edema, characterized by the macular swelling due to sub- and intra-retinal fluid accumulation resulting from iBRB breakdown, can manifest across all DR stages and remains the primary cause of vision loss in diabetic patients [162, 163, 166–168].

### EVs from retinal and circulatory sources in DR pathogenesis

**Fig. 3:**
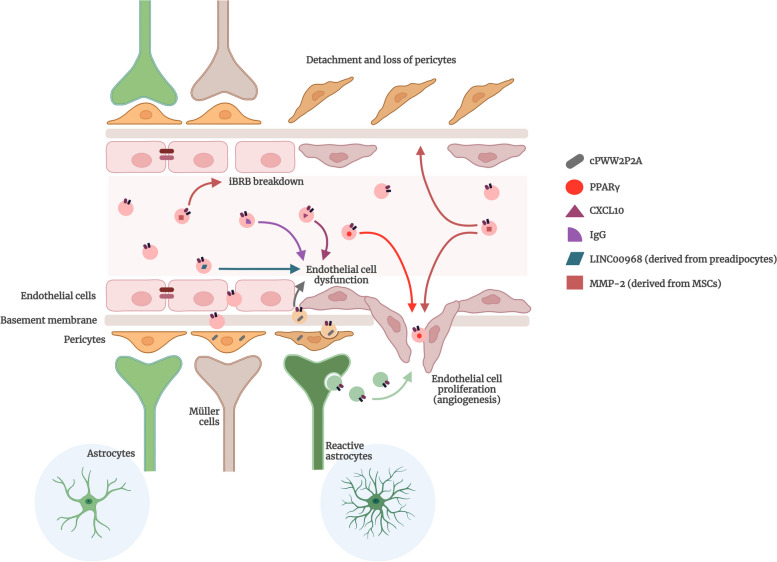
Contribution of extracellular vesicles to the pathogenesis of diabetic retinopathy. Extracellular Vesicles (EVs), both circulating EVs and retinal cells-derived EVs, play critical roles in several key processes associated with the pathogenesis of diabetic retinopathy (DR). Particularly, circulating EVs derived from mesenchymal stem cells (MSCs) that have matrix metalloproteinase 2 (MMP-2) in their cargo, are central players in the detachment and loss of pericytes, an early hallmark event in DR, which leads to microvascular dysfunction. These MMP-2-containing EVs are also known to be involved in the breakdown of the inner blood-retinal barrier (iBRB) and in the angiogenic process, both important hallmarks of DR. Peroxisome proliferator-activated receptor gamma (PPARγ)-containing EVs have also an important role in the endothelial cell proliferation. Additionally, circulating EVs containing IgG as well as preadipocyte-derived EVs positive for LINC00968 (long intergenic non-protein coding RNA 968) and EVs containing C-X-C Motif Chemokine Ligand 10 (CXCL10) contribute to endothelial cell dysfunction. Moreover, retinal cells-derived EVs, such as EVs secreted by pericytes containing cPWW2P2A and reactive astrocytes-derived EVs also contribute to the progression of DR, since they exacerbate endothelial cell dysfunction and angiogenesis thereby contributing to the disruption of retinal microvascular integrity. Created with BioRender.com

As a multifactorial condition, the pathogenesis of DR involves low grade inflammation and oxidative stress. Under normal conditions, the effective crosstalk among endothelial cells, pericytes, and glial cells is pivotal for maintaining iBRB integrity. However, early diabetes-related endothelial dysfunction leads to disruption in retinal mitochondria and metabolic pathways, disrupting intercellular communication among retinal endothelial cells, neuroglial cells, and leukocytes. This disruption can disturb retinal homeostasis, causing iBRB breakdown and increased vascular permeability [169]. EVs have recently come into focus for their potential role in the onset and progression of DR [19, 170–173]. These EVs may originate from various retinal cells or circulate systemically, entering the eye due to increased vascular permeability associated with DR [163, 174, 175]. These EVs potentially contribute to microvascular damage in the context of DR, mediating diabetes-associated pathological processes with pro-inflammatory, pro-angiogenic, and proteolytic effects (Fig. 3) [176]. For instance, a study involving patients undergoing vitrectomy revealed not only the presence of blood-derived microvesicles (such as platelet-derived and endothelium-derived microvesicles), but also vesicles positive for peanut agglutinin lectin (PNA) and isolectin B4 (IB4), indicating their photoreceptor and microglial origins, respectively, especially in patients with proliferative DR. This study further demonstrated that vitreous microvesicles isolated from DR patients stimulate endothelial cell proliferation and neovascularization [177]. Moreover, exosomes discharged by retinal cells harbor pro-angiogenic elements, whereas those from retinal astrocytes carry anti-angiogenic factors. Nevertheless, when exposed to oxidative stress, exosomes released by astrocytes have the potential to stimulate the growth and movement of endothelial cells [178]. In another study, the transfer of cPWW2P2A, a circRNA that is upregulated in retinal pericytes in DR, can be transferred from pericytes to endothelial cells through exosomes, inducing retinal vascular dysfunction [89]. Thus, the intercellular communication between retinal cells through EVs is also an important mechanism involved in DR complications, namely pericyte loss, endothelial dysfunction, and angiogenesis.

Concerning the circulating EVs from diabetic patients, a recent study reported a higher concentration of EVs in diabetic patients with DR when compared to both diabetic patients without complications and controls. Those EVs increased the detachment in human retinal pericyte cultures, the permeability of a BRB in vitro model, the migration of microvascular cells and the formation of new vessels [179, 180]. In fact, nucleic acids transfer between retinal cells is one of the most important mechanisms for disease progression.

It has been reported the increased levels of exosomes incorporating the peroxisome proliferator-activated receptor gamma (PPARγ) in the vitreous fluid and aqueous humor of diabetic patients with proliferative DR, suggesting that PPARγ may be involved in neovascularization occurring in proliferative DR [181]. Moreover, two studies from Ogata and colleagues demonstrated that both platelet-derived and monocyte-derived microvesicles are related to DR progression, being both increased in diabetic patients with DR. The number of these microvesicles increased as the stage of DR advanced, especially in patients with capillary occlusion [182, 183]. However, the role of circulating EVs of DR patients in the coagulation cascade is controversial. Some studies described the procoagulant properties of MVs in the context of DR [182, 184], but another study reported that MVs from DR patients are less procoagulant than those from diabetic patients with other vascular complications. Nevertheless, the same study also reported that these MVs exhibit higher pro-angiogenic effects, being able to induce the formation of tube networks that collapse over time [185]. Therefore, the role of circulating EVs in DR pathogenesis appears to be mainly related to their pro-angiogenic and pro-inflammatory properties (Fig. 3).

### Role of EVs on retinal vascular dysfunction and protection in diabetes

It is widely accepted that chronic (neuro)inflammation and oxidative stress have a major role in the development and progression of DR, particularly in vascular dysfunction [186–189].

The studies performed until now assessing the role of EVs on retinal endothelial cell injury in the context of diabetes/hyperglycemia have identified several molecular players with important roles in endothelial cell dysfunction (Fig. 3). For example, the levels of exosomes derived from platelet-rich plasma (PRP-Exo) are significantly increased in diabetic rats. Moreover, PRP-Exo activate the toll-like receptor-4 (TLR-4) pathway by transferring C-X-C Motif Chemokine Ligand 10 (CXCL10), increasing inflammation. The inhibition of this pathway significantly protects retinal vessels, decreasing BRB permeability in diabetic animals. When human retinal endothelial cells (HRECs) were exposed to PRP-Exo, there was an increase in the levels of malondialdehyde (MDA) and ROS, and an inhibition of the activity of superoxide dismutase (SOD), thus increasing endothelial cell injury. Those effects were inhibited by blocking the TLR4 pathway. These observations indicate that PRP-Exo mediates retinal endothelial cell injury triggered by hyperglycemia by upregulating the TLR4 pathway [190]. In another study, plasma EVs from patients with DR increased the permeability of endothelial/pericyte bilayers, induced pericyte detachment, migration of endothelial cells and pericytes, and the formation of vessel-like structures, comparing to the EVs isolated from control subjects [179]. Two studies have linked the effects of EVs with vascular damage and permeability, and with inflammation and the complement system, which are also known to have a key role in the pathophysiology of DR. IgG-laden exosomes in plasma are increased in diabetes, and these exosomes activate the classical complement pathway in mice and rat. Moreover, the lack of IgG in exosomes in diabetic mice decreases vascular damage in the retina [171, 191]. HRECs exposed to the plasma of diabetic rats results in the deposition of membrane attack complex (MAC) and cytolytic damage. The removal of EVs from plasma reduces the MAC deposition in HRECs and cytolytic damage, further supporting the important role of EVs and MAC in DR [191].

Cell-cell crosstalk mediated by EVs, even between cells that are not in close contact, can play a role in retinal endothelial dysfunction. A study explored the crosstalk between preadipocytes (3T3-L1) and mouse retina microvascular endothelial cells (mRMECs). 3T3-L1 preadipocytes treated with high glucose significantly increased the levels of LINC00968 (long intergenic non-protein coding RNA 968) in small EVs derived from these cells. Co-cultures of mRMECs with 3T3-L1 preadipocytes caused endothelial cell damage, in which inflammation and oxidative stress play a role. In the presence of high glucose, the endothelial cell damage was enhanced. Also, high glucose per se and in co-cultures, the expression of LINC00968 increased in mRMECs. Moreover, the Exo-derived from 3T3-L1 also increased LINC00968 expression in mRMECs. The inhibition of LINC00968 protected endothelial cells [192].

Mesenchymal stem cells (MSCs) are usually considered protective in many pathologies. However, if MSCs are exposed to diabetic like-conditions, EVs derived from MSCs may affect retinal vessels, interfering with the interactions between endothelial cells and pericytes. These EVs are able to enter pericytes and cause their detachment and migration, also increasing BRB permeability. In addition, EVs-derived from MSCs exposed to hyperglycemic conditions promote in vitro angiogenesis in endothelial and pericytes co-cultures, being these effects mediated by matrix metalloproteinase-2 (MMP-2), which is present not only in EVs but also expressed in pericytes [193].

In summary, these data published in the last decade highlight the importance of EVs in retinal dysfunction under hyperglycemic conditions/diabetes, and particularly the breakdown of BRB, pericyte detachment and migration, and neovascularization, processes in which inflammation and oxidative stress play a major role (Fig. 3).

### miRNA on DR pathogenesis

Several recent reports highlighted the role of miRNAs on DR progression (Table 3) [194–196]. It was demonstrated that miR-15a, produced by pancreatic β-cells, increased in plasma EVs from diabetic patients and were related with retinal injury. Pancreatic-derived miR-15a was overexpressed in Müller cells, where it was able to induce oxidative stress by targeting Akt3, leading to retinal cell injury [197]. Other study also related two angiogenic miRNAs present in circulating EVs, miR-27b and miR-320a, with the progression of DR [198]. Several miRNAs were differentially expressed when comparing EVs from controls and diabetic patients with and without DR, and the authors highlighted a potential role for miR-150-5p, miR-21-3p and miR-30b-5p, which are involved in the hypoxia-induced retinal damage, characteristic of DR, and therefore could eventually be used as prognostic biomarkers for this disease [179, 180]. Nevertheless, a recent study detected increased levels of miR-431-5p in the circulating EVs of patients with proliferative DR, that appear to be involved in the proliferation of retinal endothelial cells [199].

Several other miRNAs in EVs may be involved in DR pathogenesis. A study already mentioned in this review demonstrated that exosomes derived from preadipocytes induced damage to endothelial cells in a high glucose environment. This detrimental effect was mediated by miR-361-5p, and the study identified TNF receptor associated factor 3 (TRAF3) as a target gene of miR-361-5p. Lower levels of miR-361-5p led to overexpression of TRAF3 and induced mRMEC dysfunction in vitro [192]. Another study evaluated the effect of elevated glucose concentration on photoreceptors-derived EVs. The high glucose leads not only to increased levels of VEGF in the EVs but also to a decrease in the levels of four anti-angiogenic miRNAs (miR-20a-3p, miR-20a-5p, miR-106a-5p, and miR-20b), thus triggering a pro-angiogenic environment and the promotion of tube formation in Human Umbilical Vein Endothelial Cells (HUVECs) [200].

Altogether, these findings not only give additional insight on the pathogenesis of diabetic retinopathy, but also allows to identify potential novel targets for future therapies against this devastating disease.

**Table 3:** Key miRNAs in extracellular vesicles associated with diabetic retinopathy

miRNA	Expression level in EVs (DR phenotype)	Predicted target	Function/effect	Ref
miR-15a	upregulated	Akt3	Retinal injury and induction of oxidative stress	[197]
miR-27b and miR-320a	upregulated	Thrombospondin-1 (TSP-1)	Angiogenesis	[198]
miR-361-5p	downregulated	Tumor necrosis factor receptor-associated factor 3 (TRAF3)	Dysfunction of endothelial cells	[192]
miR-431-5p	upregulated	cAMP-specific 3’,5’-cyclic phosphodiesterase 4 A (PDE4A)	Proliferation of endothelial cells	[199]
miR-20a-3p, miR-20a-5p, miR106a-5p and miR-20b	downregulated	VEGF	Induce a pro-angiogenic environment	[200]
miR-150-5p, miR-21-3p, miR-30b-5p	upregulated	Hypoxia-inducible factor − 1 alpha (HIF-1α)	Hypoxia-induced retinal damage	[179, 180]

## Conclusions

Over the past two decades, the landscape of research in EVs has undergone a profound transformation. These tiny entities have taken center stage in research, exploring their intricate roles in disease pathogenesis and their promising utility as clinical biomarkers.

Among EVs, the small EVs, namely exosomes, have emerged as particularly captivating entities due to their remarkable capacity to ferry biomolecules between retinal cells and traverse biological barriers in both directions. The paradigm shift between mere carriers of cellular debris to a multitude of novel roles and functions, underscores the significance of EVs as key players in the intricate web of biological mechanisms. They are far more than passive actors; they are dynamic entities with the power to influence disease trajectories.

The remarkable capacity of EVs to navigate biological barriers, combined with their precise targeting capabilities and cargo delivery systems, opens up thrilling opportunities across numerous research domains and potential therapeutic realms. A deeper comprehension of the intricate mechanisms governing EVs targeting, cargo release, and their interactions with biological barriers is instrumental in unlocking the complete potential of EVs. These tiny vesicles stand as potent facilitators of intercellular communication and as versatile vehicles for therapeutic interventions in a wide array of biological scenarios, including diseases that implicate the integrity of the BRB.

In conclusion, the intricate interplay between EVs and two of the most prevalent retinal diseases, AMD and DR, reveals a compelling narrative of molecular communication within the ocular microenvironment.

In the context of AMD, EVs serve as critical messengers in the intricate molecular landscape of this complex disorder. The selective sorting of miRNAs into EVs adds a layer of complexity to their involvement. miRNAs within EVs have been implicated in processes such as autophagy, inflammation, oxidative stress, angiogenesis, apoptosis, and phagocytosis, all central to AMD pathogenesis. Notably, their influence extends beyond AMD hallmarks; EV-enclosed miRNAs have been shown to modulate the inflammatory response, offering potential targets for therapeutic intervention.

In the realm of DR, EVs have emerged as crucial mediators in the progression of this disease, governing some disease hallmarks. These tiny vesicles facilitate the dissemination of bioactive molecules that perpetuate endothelial dysfunction, iBRB breakdown, and neovascularization. Moreover, not only retinal cell-derived EVs, but also circulating EVs are both involved in orchestrating these pathological processes. Their cargo, including miRNAs, proteins, and nucleic acids, can trigger pro-inflammatory and pro-angiogenic cascades, leading to retinal damage. Moreover, recent insights into the complement activation pathway suggest that EVs play a role in the breakdown of the iBRB in DR, further solidifying their relevance in this context.

The cumulative evidence presented in this review underscores the pivotal role of EVs in the onset and progression of AMD and DR. These tiny vesicles act as potent carriers of biological information, shaping the retinal microenvironment and influencing the fate of retinal cells. Understanding the intricate mechanisms underlying EV-mediated processes holds immense promise for the development of innovative diagnostic tools and targeted therapeutic strategies for these vision-threatening diseases. As we delve deeper into the complexities of EV biology and their specific contributions to DR and AMD, we are poised to unlock novel avenues for intervention and personalized medicine in retinal disorders.

## Data Availability

All data relevant to this review are included in the text, references, tables and figures.

## Abbreviations

Alix: ALG-2-interacting protein
AMD: Age-related macular degeneration
ARF6: ADP-ribosylation factor 6
BRB: Blood-retinal barrier
C3: Complement 3
CFH: Complement factor H
CNV: Choroidal neovascularization
COX-2: Cyclooxygenase-2
CXCL10: C-X-C Motif Chemokine Ligand 10
DR: Diabetic retinopathy
ECM: Extracellular matrix
EMT: Epithelial-mesenchymal transition
ESCRT: Endosomal sorting complex required for transport
EVs: Extracellular vesicles
HDAC6: Histone deacetylase 6
HRF: Hyperreflective foci
HRECs: Human retinal endothelial cells
HSP: Heat-shock protein
HucMSC: Human umbilical cord-derived mesenchymal stem cell
HUVEC: Human umbilical vein endothelial cells
IB4: Isolectin B4
I-BAR: Inverse bin – amphiphysin
iBRB: Inner BRB
ICAM-1: Intercellular adhesion molecule 1
ILVs: Intraluminal vesicles
IL-6: Interleukin-6
IL-8: Interleukin-8
IRMAs: Intra-retinal microvascular abnormalities
JAMs: Junctional adhesion molecules
HOXC6: Homeobox C6
LC3: Microtubule-associated protein 1A/1B-light chain 3
LINC00968: Long intergenic non-protein coding RNA 968
MAC: Membrane attack complex
MCP-1: Monocyte chemotactic protein-1
MDA: Malondialdehyde
miRNA: MicroRNA
MHCII: Class II major histocompatibility complex
mtDNA: Mitochondrial DNA
MMPs: Matrix metalloproteinases
mpRPE: Mouse primary RPE
mRMECs: Mouse retina microvascular endothelial cells
MSCs: Mesenchymal stem cells
MVBs: Multivesicular bodies
PNA: Peanut agglutinin lectin
nSMase2: Neutral sphingomyelinase 2
NVU: Neuro-Vascular Unit
oBRB: Outer BRB
OCT: Optical coherence tomography
PPARγ: Peroxisome proliferator-activated receptor gamma
PRP: Platelet-rich plasma
ROS: Reactive oxygen species
RPE: Retinal pigment epithelium
PDE4A: cAMP-specific 3',5'-cyclic phosphodiesterase 4A
PDT: Photodynamic therapy
PS: Phosphatidylserine
sEVs: Small extracellular vesicles
SOD: Superoxide dismutase
T1DM: Type 1 diabetes Mellitus
TEMs: Tetraspanin-enriched microdomains
TJ: Tight junctions
TER: Transepithelial resistance
TGFß‐2: Transforming growth factor beta 2
TRAF3: TNF receptor associated factor 3
TLR-4: Toll-like receptor-4
TSG101: Tumor susceptibility gene 101
TSPAN4: Tetraspanin-4
TNF-α: Tumor necrosis factor-α
VEGF: Vascular endothelial growth factor
ZBP1: Z-DNA-binding protein 1
ZO: Zonula occludens
